# Rate adaptive pacing in people with chronic heart failure increases peak heart rate but not peak exercise capacity: a systematic review

**DOI:** 10.1007/s10741-022-10217-x

**Published:** 2022-02-09

**Authors:** H. I. Clark, M. J. Pearson, N. A. Smart

**Affiliations:** School of Science & Technology, Exercise & Sports Science, University of New England, Armidale, NSW Australia

**Keywords:** Chronic heart failure, Chronotropic incompetence, Rate adaptive pacing, Heart rate, Exercise capacity

## Abstract

Rate adaptive cardiac pacing (RAP) allows increased heart rate (HR) in response to metabolic demand in people with implantable electronic cardiac devices (IECD). The aim of this work was to conduct a systematic review to determine if RAP increases peak exercise capacity (peak VO_2_) in line with peak HR in people with chronic heart failure. We conducted a systematic literature search from 1980, when IECD and RAP were first introduced, until 31 July 2021. Databases searched include PubMed, Medline, EMBASE, EBSCO, and the Clinical Trials Register. A comprehensive search of the literature produced a total of 246 possible studies; of these, 14 studies were included. Studies and subsequent analyses were segregated according to comparison, specifically standard RAP (RAPON) vs fixed rate pacing (RAPOFF), and tailored RAP (TLD RAPON) vs standard RAP (RAPON). Pooled analyses were conducted for peak VO_2_ and peak HR for RAPON vs RAPOFF. Peak HR significantly increased by 15 bpm with RAPON compared to RAPOFF (95%CI, 7.98–21.97, *P* < 0.0001). There was no significant difference between pacing mode for peak VO_2_ 0.45 ml kg^−1^ min^−1^ (95%CI, − 0.55–1.47, *P* = 0.38). This systematic review revealed RAP increased peak HR in people with CHF; however, there was no concomitant improvement in peak VO_2_. Rather RAP may provide benefits at submaximal intensities by controlling the rise in HR to optimise cardiac output at lower workloads. HR may be an important outcome of CHF management, reflecting myocardial efficiency.

**Supplementary Information:**

The online version contains supplementary material available at 10.1007/s10741-022-10217-x.

## Introduction

Chronic heart failure (CHF) induces change in the molecular architecture of the myocardium [1, 2]. Consequently, contractility and synchronicity of systolic and diastolic function are compromised [2], posing significant problems when metabolic demand is increased. Optimal cardiac function during exercise is dependent on an ability to increase heart rate (HR) and contractility [2], to compensate for decreased filling time of the left ventricle, which ultimately reduces stroke volume [3, 4]. The relationship between HR, stroke volume, and cardiac contractility facilitating optimal cardiac hemodynamics is known as the force frequency relationship (FFR), which is intrinsic to cardiomyocytes [5–7]. In people with CHF, stroke volume adaptations to increased work and compensatory HR increases are critical to maintain adequate cardiac output [2]. At least 30–50% of people with CHF experience an inability to increase their HR to meet metabolic demands, generally termed chronotropic incompetence (CI) [8–11]. Also, approximately 25–30% of people with CHF experience electromechanical dysfunction resulting in atrioventricular, inter-ventricular, or intra-ventricular dysynchrony [8, 12–14]. This undermines the FFR and compensatory mechanisms during exercise, thus contributing to exercise intolerance [15].

Improvements in survival with optimal treatment in CHF have not been matched with improvements in health related quality of life (HRQoL) and exercise intolerance [9]. People with CHF incur persistent symptoms of shortness of breath, fatigue, and reduced functional capacity that may be associated with a blunted HR response to increased metabolic demand [11, 15]. Implantable electronic cardiac devices (IECD) have become a critical component of CHF management [16]. Rate adaptive cardiac pacing (RAP) is now available in all IECD’s and was developed to assist in restoration of a physiological HR response to an increase in metabolic demand [10]. However, RAP is controversial and not well evidenced, demonstrating mixed results among other cardiac populations [17–20]. Excessive increases in HR may lead to ischemia, decreased LV diastolic filling time, and reduced contractility in CHF [21]. Therefore, change in HR may not be a benign variable, instead constituting an important physiological treatment target in the management of CHF. It has been proposed that RAP may produce iatrogenic worsening of myocardial failure [22]. Hence, there remains incongruence on the use and programming of RAP in IECDs.

As people with CHF, particularly when encumbered with CI, observe high mortality [11] as well as high morbidity rates and hospitalisation costs due to long term systemic adaptations and complex care [23], it is important to determine if RAP provides any benefit to people with CHF [16]. The purpose of this systematic review is to determine the efficacy of RAP in people with CHF, with respect to peak HR and exercise capacity.

## Methodology

**Fig. 1:**
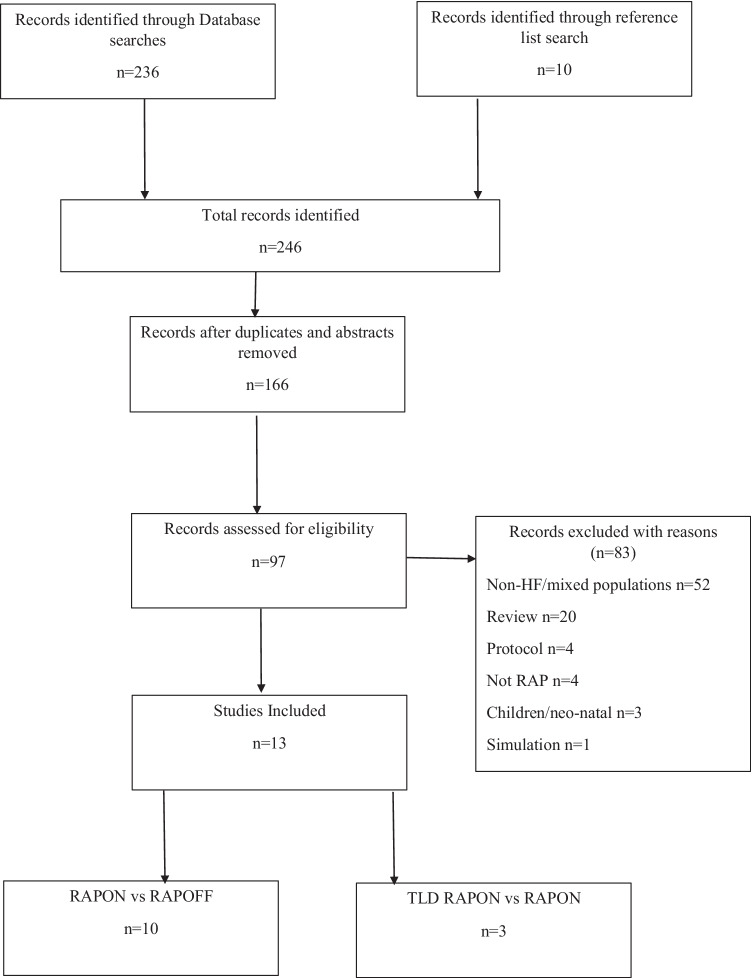
PRISMA statement

The search methodology was consistent with the PRISMA (Preferred Reporting Items for Systematic Reviews and Meta-Analyses) guidelines (Fig. 1). Reference lists of journal articles, meta-analyses, and systematic reviews were searched for additional articles. The clinical trial registry was also searched for unpublished results.

### Eligibility

#### Inclusion criteria

Studies included were those which enrolled people with CHF with heart failure with reduced ejection fraction (HFrEF) and heart failure with preserved ejection fraction (HFpEF). Studies were required to compare standard rate adaptive pacing (RAPON) with fixed rate pacing (RAPOFF) (RAPON vs RAPOFF) or tailored RAP (TLD RAPON) with standard rate adaptive pacing (RAPON) (TLD RAPON vs RAPON). Segregating these studies from RAPON vs RAOFF studies is required as the purpose of RAP differs. Specifically, TLD RAPON vs RAPON studies aim to determine if tailoring RAP achieves better outcomes compared to conventional, non-specific RAP.

Included studies were randomised controls trials, observational or cross-over designs, or unpublished clinical trials. IECD could include CRT, PM, ICD, or a combination. All modes, sensors, and algorithms were included. Studies were required to assess exercise capacity via a cardiopulmonary exercise test or the 6 min walk test (6MWT). Studies had to be full text and in English.

#### Exclusion criteria

Studies were excluded if they were not specific to CHF or did not assess symptom limited exercise capacity. Abstracts and studies in a language other than English were excluded.

### Outcomes

Primary outcome variables included peak HR, peak oxygen consumption (peak VO_2_), exercise time during maximal exercise test (ET), and 6MWT.

### Search strategy

A systematic literature search was conducted from 1980 until January 30 2021. Databases searched include PubMed, Medline, EMBASE, EBSCO, and the Clinical Trials Register. To identify studies, the following MeSH terms were used; ‘Rate Adaptive Pacing’, ‘Heart Failure’, ‘Humans’, ‘Heart Rate’, ‘Chronotropic Incompetence’, ‘Exercise Testing’, ‘Exercise Capacity’, ‘Exercise Intolerance’, ‘Cardiac Resynchronisation Therapy’, ‘Pacemaker Therapy’, and ‘Implantable Cardio-Defibrillator’ (See Supplementary File).

### Data extraction and study selection

One researcher (HC) extracted the data which was validated by a second researcher (MJP). Inconsistencies were reviewed by a third researcher (NAS) and resolved through consensus. This process was undertaken to ensure reliability and reduce the risk of bias. Article titles were screened for assessment of RAP. Abstracts and methodology were then reviewed according to the inclusion/exclusion criteria. All duplicates were removed.

### Calculating confidence intervals

In this review, 95% and 84% confidence intervals (CONI) were calculated for each mean value where appropriate. The test of overlapping CONI when comparing an intervention to a control can be conducted to assess if there is a true statistical difference between the means. Mittal et al. [24] and Austin and Hux [25] suggest that if the CONI overlap at the 95% level, it does not necessarily refute the true statistical difference between the means. However if they do not overlap, then the statistical significance cannot be doubted. Alternatively, if one calculates the CONI at the 84% level, if the intervals do not overlap, the *P* value associated with testing the difference between the two means is approximately 95% (*P* = 0.05) and is likely statistically different from one another [25, 26]. Furthermore, MacGregor-Fors and Payton [26] demonstrate that this test holds true for both asymmetric and symmetric CONI, reinforcing the robustness of this method.

To calculate each confidence interval, the standard deviation (SD) was multiplied by 1.96 (95%CONI) and 1.37 (84% CONI). This value was then subtracted and added to the mean value to define the lower and upper limit of the CONI, respectively [26].

### Data pooling

Data pooling occurred where appropriate, with a minimum of three studies reporting on the same outcome required. To pool data, all included studies followed the same study design, whether that be RCT or cross-over trials. When the standard error of the mean was present, the SD was calculated by multiplying the standard error of the mean by the square root of the number of participants [27]. Data evaluation was conducted using RevMan 5 (Review Manager Version 5.4.1, The Cochrane Collaboration, 2020). A random effects model was applied to account for the large variation in heart failure subtype and pacing modes, with a CONI of 95%.

### Risk of bias

Risk of bias was assessed according to the Revised Cochrane risk of bias tool for randomised trials (RoB2) with additional considerations for cross over trials [28]. The tool includes five domains, with an extra domain specific to crossover trial designs. Each question was answered with a yes (Y), probably yes (PY), not sure (NI), probably no (PN), or no (N). Each domain followed an algorithm according to the answers to the signalling questions. Domains could then be rated ‘low risk’, ‘moderate risk’, or ‘high risk’. Studies were considered low, moderate or high risk according to the greatest number of domains with the same results.

## Results

### Search

A comprehensive search of the literature produced a total of 246 possible studies. Database searches collectively returned 236 hits, while 9 papers were identified through hand searching of reference lists and one study through the clinical trials database. A total of 80 duplicates were removed, and 166 items were screened by title. Of these, 97 studies were considered for further review that included screening of abstract, methodology, and results. Subsequently, 84 studies did not meet the inclusion criteria. A total of 13 studies are included in this review.

### Risk of bias

Results for risk of bias are reported in the supplementary file. The risk of bias for randomised control trials [6, 12, 21, 29] was low across all domains for all studies. The risk of bias for crossover trials [5, 22, 30–36] was low across all domains for all studies except Van Thielen et al. [5]. Van Thielen et al. [5] recorded high risk in domain 1 as the group allocation sequence was not randomised. Additionally, moderate risk of bias is reported for domain ‘S’ due to the inability to determine equal allocation of participants and the accounting for period effects in the analyses. However, as four out of the six domains reported low risk, this study was concluded to have a low overall risk of bias.

## Rate adaptive pacing vs fixed rate pacing

### Study characteristics

Ten studies compared standard RAP (RAPON) with fixed rate pacing (RAPOFF) (Table 1) [5, 12, 22, 29, 31–36]. Two studies were randomised control trials [12, 29], while the remaining eight were cross over trials. In particular, the study conducted by Kass and colleagues [31] was suspended due to poor recruitment. Therefore, the published protocol is cited here, while unpublished results are available at Clinicaltrials.gov (NTC00670111) and included in this review.

**Table 1:** Pacemaker mode terminology

The Heart Rhythm Society and British Pacing Electrophysiology Group guide to pacemaker modes of operation				
*I*	*II*	*III*	*IV*	*V*
*Chamber(s) paced*	*Chamber(s) sensed*	*Mode(s) of response*	*Programmable functions*	*Anti-tachycardia function*
V-Ventricle	V-Ventricle	T-Triggered	R-Rate Adaptive/Responsive	O-None
A-Atrium	A-Atrium	I-Inhibited	C-Communicating	P-Paced
D-Dual (A&V)	D-Dual (A&V)	D-Dual Triggered/Inhibited	M-Multi-programmable	S-Shocks
O-None (S-Single)	O-None (S-Single)	O-None	P-Simple Programmable O-None	D-Dual (P&S)

Table adapted from DeForge [17]

**Fig. 2:**
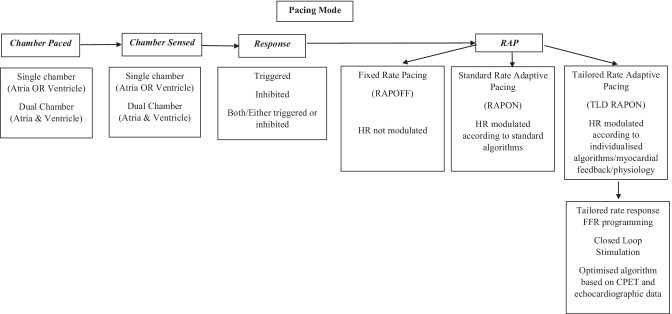
Schematic diagram of pacing modes and difference between fixed rate pacing, rate adaptive pacing, and tailored rate adaptive pacing. Cardio-Pulmonary Exercise Test (CPET); Force Frequency Relationship (FFR); Heart Rate (HR)

Across studies, devices included single chamber, dual chamber, and three chamber PM/ICD/CRT and included atrial, left uni-ventricular, and bi-ventricular (BiV) pacing. Modes included VVI, DDI, DDD, and AAI, with or without RAP (R) (Fig. 2: Schematic diagram of pacing modes; Table 2: Pacemaker Modes Terminology). RAP sensors included accelerometers [21, 30, 32, 33, 36] and blended minute ventilation and accelerometers [31]. Six studies did not specify the sensor used [5, 12, 22, 29, 34, 35].

**Table 2:** Characteristics of included studies standard RAP (RAPON) vs fixed rate pacing (RAPOFF)

Study (year)	Sample size	HF type	Rhythm	CI criteria	Device type	Sensor	Pacemaker mode (RAPON/RAPOFF)*	RAPON	RAPOFF
Jamil et al. [22]	79	HFrEF	SR (53) AF (26)	Chronotropic index < 0.80	CRT PM ICD	Unknown sensor	> 95% BiV pacing (CRT) 0% ventricular pacing (non CRT)	RAPON	RAPOFF
Kass et al. [31]	13	HFpEF	SR	≤ 80% HRR ≤ 62% HRR in those tx BB	Implantable cardiac device	Blended minute ventilation and accelerometer	AAIR/AAI	AAIR	AAI
Palmisano et al. [32]	60	HFrEF with drug refractory AF	AVJ Ablation and BiV pacing	100% iatrogenic CI after AV nodal ablation	CRT	Accelerometer	VVIR/VVI	VVIR	VVI
Passman et al. [33]	10	HFrEF	SR	No CI Criteria	Dual chamber ICD	Accelerometer/Crystal	AAIR/VVI	AAIR	VVI
Pu et al. [12]	72	HFrEF	SR	No CI Criteria	Three chamber CRT-PM/ICD Dual chamber PM	Unknown Sensor	RAAVD LUV/BiV	RAAVD LUV (RAP-ON)	BiV Pacing (RAP-OFF)
Shanmugam et al. [34]	20	HF	SR	No CI Criteria	CRT-PM/ICD	Unknown sensor	RAAVD LUV/BiV	RAAVD-ON	BiV Pacing
Sims et al. [35]	13	HFrEF	SR	< 70% APMHR	CRT	Unknown sensor	DDDR/DDD	DDDR	DDD
Tse et al. [36]	20	HFrEF	SR	< 70% APMHR (*n* = 11)/70%–85% APMHR (*n* = 9)	CRT-PM (*n* = 7) CRT-ICD (*n* = 3) CRT (*n* = 10)	Accelerometer	DDDR/DDD	DDDR	DDD
Van Thielen et al. [5]	14	HFrEF	SR	< 85% APMHR	CRT	Unknown sensor	DDDR/DDD	DDDR	DDD
Zhao et al. [29]	60	HFrEF	SR	No CI Criteria	Three-chamber PM Dual chamber PM	Unknown sensor	RAAVD LUV/BiV	RAAVD	BiV pacing

*AF* atrial fibrillation, *APMHR* age predicted maximum heart rate, *AV* atrio-ventricular, *AVJ* atrio-ventricular junction, *BB* beta-blockers, *BiV* biventricular, *CI* chronotropic incompetence, *CRT* cardiac resynchronisation therapy, *HF* heart failure, *HFpEF* heart failure preserved ejection fraction, *HFrEF* heart failure reduced ejection fraction, *HRR* heart rate reserve, *ICD* implantable cardio-defibrillator, *LUV* left univentricular, *PM* pacemaker, *RAAVD* rate adaptive atrioventricular delay, *RAP* rate adaptive pacing, *RAPOFF* fixed rate pacing, *RAPON* standard rate adaptive pacing, *SR* sinus rhythm

### Participant characteristics

**Fig. 3:**
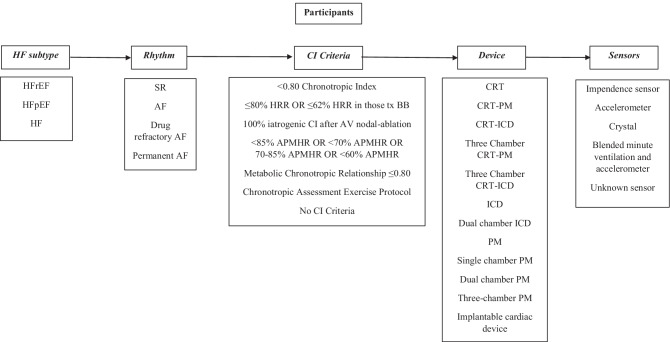
Overview of participant characteristics across all included studies. Atrial fibrillation (AF), age predicted maximum heart rate (APMHR), atrio-ventricular (AV), beta-blockers (BB), chronotropic incompetence (CI), cardiac resynchronisation therapy (CRT), heart failure (HF), heart failure preserved ejection fraction (HFpEF), heart failure reduced ejection fraction (HFrEF), heart rate reserve (HRR), implantable cardio-defibrillator (ICD), pace maker (PM), sinus rhythm (SR)

An overview of study inclusion criteria and the variation in participant characteristics can be viewed in Fig. 3. The mean age of participants ranged from 54 [29] to 76 years [22]. A larger proportion of males was reported across studies (63.3 [32] to 90% [33]). Ischemic aetiology was prominent across HFrEF cohorts (13.3 [29]to 80% [32]). Additionally, hypertension was present across a similar spectrum (28% [22] to 86.7% [32]). Beta blockers (BB) and ACE inhibitors/angiotensin receptor blockers (ACEI/ARBS) were widely prescribed across the participants. Beta-blockers were most common at 82 [36] to 100% [5, 33] and ACEI/ARBs at 32.8 [22] to 100% [35] of participants, respectively. All four New York Heart Association (NYHA) classes were represented across the studies.

All but two studies [31, 34] identifiably reported on people with HFrEF. The mean left ventricular ejection fraction (LVEF) in people with HFrEF across the studies was reported from 17 [35] to 39.7% [32]. Those with HFpEF had a LVEF > 50% [31], while Shanmugam et al. [34] in people of unknown heart failure phenotype reported a mean LVEF of 42.5%. Two studies included participants with atrial fibrillation (AF). Specifically, Jamil et al. [22] included a separate group analysis of 26 participants with AF, while Palmisano et al. [32] included 60 participants with drug refractory permanent AF undergoing atrio-ventricular junction ablation. Six out of ten studies defined CI but in various ways [5, 22, 31, 32, 35, 36].

### Outcomes

Data are presented below and in the supplementary file. Data are reported as mean (95% confidence interval, *P* value for test of significant difference).

#### Peak heart rate

**Fig. 4:**
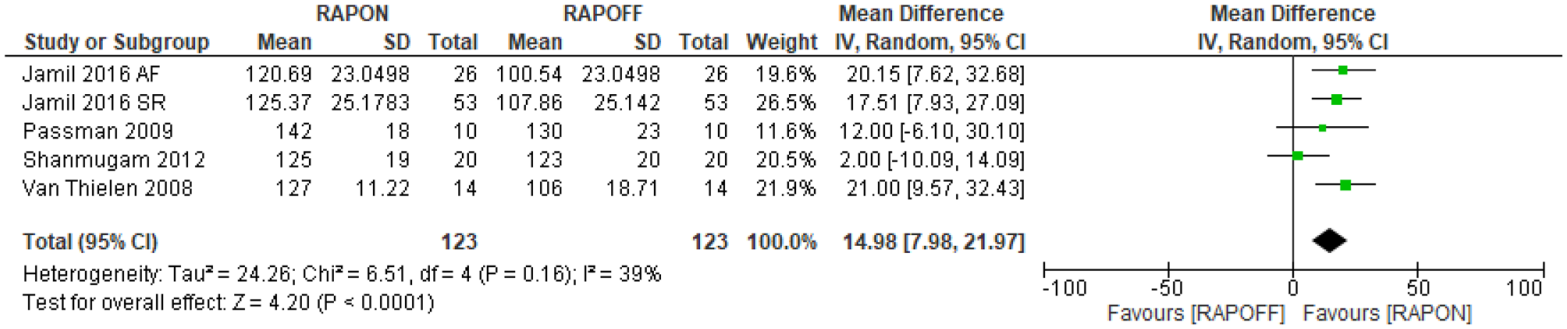
Peak HR RAPON vs RAPOFF

Four studies assessed peak HR while undergoing cardiopulmonary exercise testing [5, 22, 33, 34]. A total of 123 participants were included in the RAPON group and RAPOFF group, respectively. The data supported RAPON in increasing peak HR compared to RAPOFF. The mean difference was 14.98 bpm (7.98, 21.97, *P* < 0.0001), and level of heterogeneity was moderate (*I*^2^ = 39%) (Fig. 4).

All studies demonstrated an increase in peak HR with RAPON compared to RAPOFF, with statistically significant differences established in three studies: Jamil et al. [22] in both HFrEF and AF and HFrEF and SR groups, and Passman et al. [33] and Van Thielen et al. [5] in HFrEF and SR participants. Both Jamil et al. [22] and Van Thielen et al. [5] demonstrated no CONIs overlap at the 95% level, while all other CONI overlapped (supplementary file).

#### Peak oxygen uptake (peak VO_2_)

**Fig. 5:**
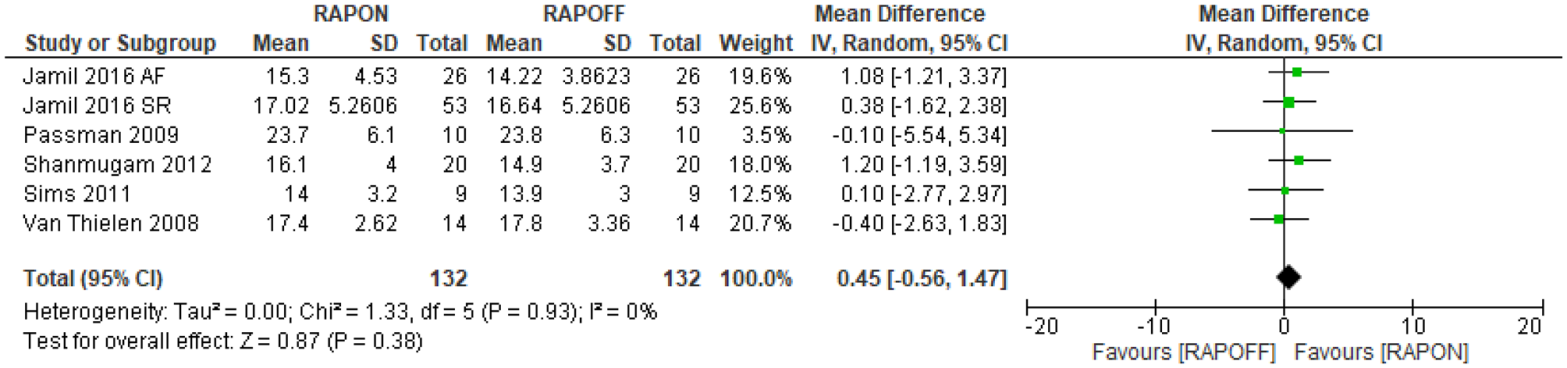
Peak VO_2_ RAPON vs RAPOFF

Six studies assessed peak VO_2_ [5, 22, 31, 33–35]; however, only five are included in the pooled analysis due to the unpublished nature of Kass et al. [31]. There were a total of 132 participants in the RAPON and RAOFF groups, respectively. The data supported a lack of effect of RAPON on peak VO_2_. The mean difference was 0.45 ml kg^−1^ min^−1^ (− 0.55, 1.47; *P* = 0.38), level of heterogeneity was low (*I*^2^ = 0%) (Fig. 5).

Only two studies demonstrated a significant increase in peak VO_2_ with RAPON compared to RAPOFF. Jamil et al. [22] demonstrated a significant increase in peak VO_2_ in the HFrEF and AF group; however, this was not reflected in the HFrEF and SR group. Shanmugam et al. [34] observed a significant increase in peak VO_2_ with RAAVD compared to standard BiV pacing. All CONIs overlapped at the 95% and 84% levels (supplementary file).

#### Exercise time

**Fig. 6:**
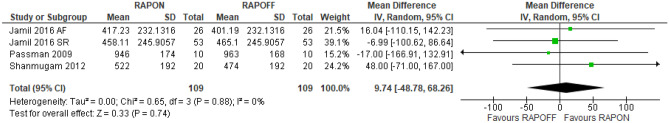
ET RAPON vs RAPOFF

Three studies assessed ET during peak VO_2_ assessment. All were included in the pooled analysis. There were a total of 109 participants in the RAPON and RAPOFF groups. The data demonstrated a non-significant increase in ET with RAPON compared to RAPOFF. The mean difference was 9.74 s (− 48.78, 68.26; *P* = 0.74); level of heterogeneity was low (*I*^2^ = 0%) (Fig. 6).

Only one study demonstrated a significant increase in ET with RAPON compared to RAPOFF. Shanmugam et al. [34] observed an increase in ET of 48 s in people with HF in SR with RAAVD compared to standard BiV pacing. All CONIs overlapped at the 95% and 84% levels (supplementary file).

#### Six-min walk test

Four studies assessed exercise capacity using 6MWT [12, 29, 32, 35]. Differing study designs did not permit pooled analysis. All studies demonstrated an increase in the 6MWT with RAPON compared to RAPOFF. However, only two studies demonstrated a significant increase in distance including Palmisano et al. [32] (HFrEF and atrioventricular junction ablation for AF) and Sims et al. [35] (HFrEF and SR). All studies demonstrated CONIs crossed over at both the 95% and 84% levels (supplementary file).

## Tailored rate adaptive pacing vs standard rate adaptive pacing

### Study characteristics

Three studies compared tailored RAP programming (TLD RAPON) with standard RAP (RAPON) (Table 3) [6, 21, 30]. Two studies were randomised control trials [6, 21]. Devices included single or dual chamber PM, CRT, and ICD. Modes include VVIR, DDDR, and AAIR. One study employed a closed loop stimulation/impedance sensor in the TLD RAPON group comparing it to a standard accelerometer [30] (RAPON). Serova et al. [21] utilised a PM with an accelerometer. Gierula et al. [6] did not identify the sensor use.

**Table 3:** Characteristics of included studies: tailored RAP (TLD RAPON) vs standard RAP (RAPON)

Study (year)	Sample size	HF type	Rhythm	CI criteria	Device type	Sensor	Pacemaker mode (RAPON Tailored/RAPON Standard)*	TLD RAPON	RAPON
Gierula et al. [6]	83	HFrEF	SR(53) AF(30)	No CI criteria	CRT or ICD	Unknown sensor	VVIR (AF) DDDR (CRT) AAIR (no CRT) DDDR (no CRT + long AVD)	Tailored rate response FFR programming	Conventional rate response programming
Hsu et al. [30]	12	HFrEF	SR	Metabolic chronotropic relationship ≤ 0.80	CRT-ICD	Impendence sensor/standard accelerometer	DDDR/CLS	Closed loop stimulation	Standard DDDR pacing
Serova et al. [21]	54	HFpEF with permanent AF	AF	Chronotropic assessment exercise Protocol^84^	Single chamber PM	Accelerometer	VVIR	Optimised algorithm based on CPET and echocardiographic data	Conventional age-based programming

*AF* atrial fibrillation, *APMHR* age predicted maximum heart rate, *CI* chronotropic incompetence, *CLS* closed loop stimulation, *CPET* cardio-pulmonary exercise training, *CRT* cardiac resynchronisation therapy, *FFR* force frequency relationship, *HF* heart failure, *HFpEF* heart failure preserved ejection fraction, *HFrEF* heart failure reduced ejection fraction, *ICD* implantable cardio-defibrillator, *PM* pacemaker, *RAP* rate adaptive pacing, *RAPON* standard rate adaptive pacing, *SR* sinus rhythm, *TLD RAPON* tailored rate adaptive pacing

Of the three studies comparing tailored RAP (TLD RAPON) to standard RAP (RAPON) (Table 2), Gierula et al. [6] compared a tailored rate response force-frequency-relationship (FFR) algorithm (TLD RAPON) with standard rate response programming (RAPON). Hsu et al. [30] compared closed loop stimulation (CLS/impedance sensor) (TLD RAPON) vs standard DDDR mode (accelerometer sensor) (RAPON). While Serova et al. [21] compared an optimised algorithm for RAP based on CPET and PM stress echocardiography data (TLD RAPON) with conventional RAP programming (RAPON).

### Participant characteristics

Two studies reported on people with HFrEF, with the mean LVEF 35.2 [6] to 37% [30]. Serova et al. [21] reported a LVEF of 51–53% in people with HFpEF. All studies included participants with AF including Hsu et al. [30] (18% paroxysmal AF), Gierula et al. [6] (36% unspecified AF), and Serova et al. [21] (100% permanent AF). Two the studies defined CI [21, 30]. NYHA classes I–III are represented across the studies.

The mean age of participants ranged from 69 [30] to 74 years [6]. Serova et al. [21] conducted an HFpEF study, reporting a higher proportion of females (54.5%). Males made up 56 [30] to 71% [6] in studies including people with HFrEF. Ischemic aetiology was presented in 63% of HErEF participants [6]. While hypertension was more prevalent in people with HFpEF (86% [21] vs 47% [6]). BB and ACEI/ARBS were widely prescribed across the participants. BB were most common at 86 [21] to 96% [6] and ACEI/ARBs at 77 [21] to 94% [6] of participants, respectively.

### Outcomes

Differences in study design (2 randomised control trials; 1 cross over trials) did not allow for data pooling. Data from all studies is presented in the supplementary files.

#### Peak HR

Two studies comparing TLD RAPON vs RAPON modes reported peak HR while undergoing CPET [6, 30]. There was no significant difference between peak HR values for TLD RAPON vs RAPON reported in any study. All CONI overlapped at the 95% and 84% levels.

#### Peak oxygen uptake (peak VO2)

All three studies comparing TLD RAPON vs RAPON modes reported peak VO_2_ [6, 21, 30]. Two studies [6, 30] showed no improvement in peak VO_2_ with TLD RAPON vs RAPON. Only Serova et al. [21] demonstrated an increase in peak VO_2_ in people with HFpEF and AF with an optimised algorithm tailored to participants individual CPET and PM stress echocardiography data compared to standard RAPON programming. All CONIs overlapped at the 95% and 84% levels.

#### ET

Two studies assessed ET during peak VO_2_ assessment. Both studies demonstrate a significant increase in ET with TLD RAPON compared to RAPON. Gierula et al. [6] observed an increase in ET of 81 s (*P* = 0.044) in people with HFrEF in SR or AF, while Serova et al. [21] observed an increase in ET of 147 s (*P* < 0.0001) in people with HFpEF and AF. All CONIs overlapped at the 95% and 84% levels (supplementary file).

#### Six-minute walk test

Serova et al. [21] was the only TLD RAPON vs RAPON study to assess 6MWD. There was no significant change between tailored and standard RAP with CONIs demonstrating cross over at the 95% and 84% levels.

## Discussion

We conducted a systematic review and data synthesis of studies comparing the effects of RAP on exercise capacity in people with CHF. RAP is largely indicated for people with CI, which is prevalent in 30–50% of people with CHF [8, 11]. We separated our analyses into studies comparing RAPON vs RAPOFF and those comparing TLD RAPON vs RAPON. The main findings from this review are that it is evident that RAPON increases peak HR in people with CHF, the key feature of this modality. Although, the increase in peak HR occurs without a concomitant improvement peak VO_2_, suggesting that CI is not related to exercise capacity in people with CHF [22, 37]. This is contrary to the positive relationship between HR and VO_2_ in healthy individuals. Consequently, there seems to be an uncoupling of peak HR and peak exercise capacity in people with CHF [38].

### Heart rate

Heart rate acutely regulates myocardial contractile state and subsequently cardiac output. The force frequency relationship (FFR) is intrinsic to human cardiomyocytes and governs that an increase in HR increases contractile function [39]. The intrinsic molecular integration of cardiomyocytes facilitates an increase in acceleration of shortening and re-lengthening that optimises filling time and increases systolic force with shortened cycle length. This positive FFR is critical to maintaining adequate cardiac output to meet metabolic demands [29, 39, 40]. However, the FFR is impaired in senescent human cardiomyocytes that may parallel the physiological decrement in peak HR with age [39, 40].

The FFR is especially diminished in diseased myocardium, suggesting that higher peak HRs in people with CHF may not be conducive to the underlying pathophysiology [41]. Although CI in people with CHF is associated with increased HF hospitalisation and mortality [11], it has been suggested that CI may be a compensatory mechanism to avoid an increase in myocardial work at the expense of cardiac output [22]. Limiting the maximum HR achievable is important to minimise ischemia [42, 43], myocyte apoptosis [44, 45], and cardiac remodelling [46]. For instance, Gierula and colleagues [6] tailoring RAP (TLD RAPON) according to individuals FFR significantly reduced peak HR by 13 bpm when compared to RAPON. This occurred in the setting of increased exercise time (ET) without a concomitant increase in peak VO_2_. This indicates that a lower exercise HR, facilitating an optimal FFR in people with CHF, may promote an increase in work output without increasing oxygen demands. A consequence of increased myocardial efficiency and optimal cardiac output [6, 7, 47].

Due to central and peripheral limitations, people with CHF rely disproportionately on their HRR to augment cardiac output and thus peak VO_2_. Therefore, HRR demonstrates strong prognostic value in this population [48, 49]. Bangalore et al. [49] showed that %HRR was a good measure of CI and has further prognostic value than 85%APMHR and other standard cardiovascular risk factors. In addition, %HRR was also independent of echocardiograpically determined myocardial ischemia and LVEF, and was an independent predictor of cardiovascular events. As HRR is determined by the difference between resting and peak HR, this measure may be more applicable to the FFR, representing a shift down the curve [50]. Therefore, people with CHF have a reduced intrinsic myocardial capacity for increased contractility [51].

The importance of enhancing HRR in people with CHF is reflected in prescription of beta-adrenergic blocking (BB)-agents as cornerstone therapy [52]. CHF pathology is associated with sympathetic overdrive that leads to a down regulation and desensitisation of beta-adrenergic receptors [53–55]. BB-prescription is associated with the subsequent upregulation of beta-adrenergic receptors that might conserve inotropy and chronotropy by lowering resting HR [56, 57]. A lower resting HR and greater HRR is correlated with improvements in exercise capacity, central hemodynamics, and prognosis in people with CHF [58]. Carvalho and colleagues [59] demonstrated that the relationship between the %VO_2_ reserve and %HRR in CHF patients on optimised BB-therapy was reliable, but this relationship was unreliable in non-optimised CHF patients. This indicates an intrinsic link between reducing myocardial work, improving adrenergic sensitivity and contractility [60]. Also, increased myocardial work at lower intensities may reflect an impaired FFR such that higher HRs are required to maintain adequate cardiac output at reduced metabolic loads. Thereby improving adrenergic sensitivity and increasing vagal tone may consequently improve filling time and systolic force reducing myocardial work for any given intensity. This is reflected in studies on calcium handling in failing and non-failing cardiomyocytes [39, 40, 61, 62]. Efficient calcium handling is critical to maximising filling time with increasing HRs that in turn enhances systolic function. Senescent and failing cardiomyocytes exhibited prolonged calcium handling dynamics with increased pacing frequency which correlated with impaired cell shortening and re-lengthening frequency [39]. Therefore, significantly reducing resting HR may allow for increased filling time and improved myocardial efficiency [62]. For example, Bagriy et al. [63] demonstrated that adding ivabradine to carvedilol therapy in people with CHF significantly lowered resting HR and improved 6MWT and LVEF compared to people with CHF and carvedilol therapy alone.

### Exercise capacity

#### Peak exercise capacity

Peak exercise capacity did not improve with RAP compared to fixed rate pacing in people with CHF. Possible limiting factors of exercise capacity in people with CHF and IECD with RAP are presented by the Fick equation. It can be postulated that as there was no observed change in peak VO_2_, then arterio-venous difference limits oxygen consumption [64, 65]. Alternatively, cardiac output may be similar in RAPON compared to RAPOFF due to the inability of the failing myocardium to produce an increase in stroke volume at higher HRs [2, 38].

The stunting of exercise capacity in people with CHF and RAP may be due to unfavourable peripheral hemodynamics and skeletal muscle function [15]. Van Thielen et al. [5] observed an increase in cardiac output and peak HR without a concomitant improvement in peak VO_2_, suggesting a peripheral limitation in exercise capacity. In contrast, it has been proposed that CI in people with CHF may be an important compensatory mechanism to ensure cardiac output is not overtly diminished compared to metabolic requirements [10]. Higher peak HRs in people with CHF may negatively impact the FFR, compromising cardiac output, limiting exercise capacity [66]. Kinderman and colleagues [38] demonstrated that the optimal pacing rate for oxygen uptake in people with CHF is significantly lower than in people with normal left ventricular function, specifically, 75%APMHR. However, as no other studies in this review besides Van Thielen et al. [5] measured cardiac output or an echocardiographic surrogate at peak exercise, this hypothesis cannot be corroborated.

#### Submaximal exercise capacity

Peak VO_2_ may not be the most clinically relevant outcome of RAP in people with CHF. Some have argued that peak CPET in people with CHF is unnecessary [67–69]. The level of exertion required to validate the test is unable to be met in this population due to a number of limiting factors such as CI itself, ventilation rate, pulmonary congestion, and leg fatigue [10, 70]. Alternatively, the 6MWT may be more relevant to assess capacity to perform activities of daily living, holding greater relevance when assessing RAP [67, 71]. In this review, all studies comparing RAPON vs RAPOFF demonstrated a trend towards an improvement in 6MWT [12, 29, 32, 35] and two studies observed a significant increase [32, 35]. This may reflect the rate control of RAP, which mimics a more physiological rise in HR in the early stages of exercise, compared to a standard linear increase in RAPOFF. This may facilitate optimal cardiac output at submaximal workloads. Sims et al. [35] demonstrated clearly the early rise in HR at the commencement of exercise facilitated by RAPON compared to RAPOFF during a graded maximal exercise test. This early rise in HR may have greater relevance for submaximal exercise, enabling a greater work output without increasing VO_2_ by optimising cardiac dynamics further down the FFR curve [7, 35, 47]. Additionally, Palmisano et al. [32] observed a significant increase in the 6MWT in RAPON mode compared to RAPOFF mode, with HR 30 bpm higher while systolic blood pressure was 9 mmHg lower for RAPON vs RAPOFF, respectively. This suitably reflects an optimal FFR such that HR increase does not exceed the critical point at which contractility and stroke volume diminish. Thus, contractility and cardiac output may be optimal with RAP at submaximal intensities which could have important implications for activities of daily living [72].

### Limitations

Studies included in this review had variation among CI criteria, pacing types, modes, and programming that may reduce an observable impact of RAP [1, 73, 74]. For example, Serova et al. [21] only included participants with HFpEF in AF with single chamber PM in VVIR mode with an accelerometer. Importantly, VVI(R) pacing has been shown to have lesser improvements over dual chamber and BiV pacing in terms of improvement in functional status and HF symptoms, dysynchrony, and cardiac remodelling. Similarly, the presence of RV pacing presents an issue when interpreting results of pacing studies as it induces dysynchrony and enhances LV dysfunction [75, 76]. Additionally, combined sensors such as blended minute ventilation and accelerometers have been shown to have superior HR modulation compared to single sensors [77, 78]. Therefore, care should be taken before extrapolating these results to people with HFrEF in SR, and those with PM in DDDR mode, CRT devices, or PM with other types of activated sensors. Also, the long-term effects of RAP are yet to be elucidated. Only one study to date has conducted long-term follow-up of people with CHF and RAP, with non-applicable results due to RV pacing [79].

### Future research

RAP may have important applications beyond attenuating CI [21]. Exercise training (ET) in people with CHF has been shown to have positive central and peripheral adaptations with minimal adverse events [80–82]. Therefore, future research should the assess the effects of RAP in ET in people with CHF and IECD, including long-term follow-up to ascertain if RAP is in anyway detrimental in this population. Additionally, as RAP may have greater implications at submaximal intensities, future research should ascertain if RAP improves efficiency by lowering VO_2_ for a given workload.

## Conclusion

The results of this systematic review confirm that RAP increases peak HR in people with CHF, however there is no concomitant improvement peak VO_2_. Therefore, CI may not be associated with exercise intolerance in people with CHF. Rather HRR, as an indication of a reserve in myocardial contractility, rather than peak HR, may be of greater importance in people with CHF. Consequently, people with CHF have an impaired FFR that may be optimised by lowering resting HR rather than inducing an increase in peak HR. Additionally, HR at rest and at submaximal intensities may be an important indicator of myocardial efficiency, indirectly reflecting patients’ FFR, providing valuable information for CHF management. In the case of RAP, its application may be better suited at modulating the rise in HR at submaximal intensities rather than increasing peak HR.

## Supplementary Information

Below is the link to the electronic supplementary material.

Supplementary file1 (DOCX 31 kb)

## Data Availability

All data is available in the supplementary file.
