# Supplementary material for: Rate adaptive pacing in people with chronic heart failure increases peak heart rate but not peak exercise capacity: a systematic review
Source: Heart Fail Rev. 2022 Feb 9;28(1):21–34. doi: 10.1007/s10741-022-10217-x (PMC9902309; doi:10.1007/s10741-022-10217-x)
Supplement: Supplementary file 1 — Supplementary file1 (DOCX 31 kb) [file 10741_2022_10217_MOESM1_ESM.docx]

***Supplementary File***

Table S1 RAPON vs RAPOFF

| Study | Population & Rhythm | Device | Mode  (RAPON/RAPOFF)* | RAP ON  *Mean (SD)* | RAP OFF  *Mean (SD)* | P | 95% Overlap | 84% Overlap |
| --- | --- | --- | --- | --- | --- | --- | --- | --- |
| **Peak HR (bpm)** | | | | | | | | |
| Jamil SR | HFrEF SR | CRT, PM, ICD | >95% BiV pacing (CRT)  0% ventricular pacing (non CRT) | 125.37  (95%CI: 118.43-132.30) | 107.86  (95%CI: 100.93-114.79) | <0.0001 | N* | N/A |
| Jamil AF | HFREF AF | CRT, PM, ICD | >95% BiV pacing (CRT)  0% ventricular pacing (non CRT) | 120.69  (95%CI: 111.38-130.00) | 100.54  (95%CI: 91.23-109.85) | 0.0008 | N* | N/A |
| Passman | HFrEF SR | Dual chamber ICD | AAIR/VVI | 142 (18)  (95%CI: 106.72-177.28)  (84%CI: 117.34- 166.66) | 130 (23)  (95%CI: 84.92-175.08)  (84%CI: 98.49-161.51) | 0.05 | Y | Y |
| Shanmugam | HF SR | CRT-PM/ICD | RAAVD/BiV | 125 (19)  (95%CI: 87.76-162.24)  (84%CI: 98.97-151.03) | 123 (20)  (95%CI: 83.8-162.2)  (84%CI: 95.6-150.4) | 0.30 | Y | Y |
| Van Thielen | HFrEF SR | CRT | DDDR/DDD | 127 (3)  (95%CI: 121.12-132.88)  (84%CI: 122.89-131.11 | 106 (5)  (95%CI: 96.2-115.8)  (84%CI: 99.15-112.85) | 0.001 | N* | N* |
| **Peak VO_2_ (ml/kg/min)** | | | | | | | | |
| Jamil SR | HFrEF SR | CRT, PM, ICD | >95% BiV pacing (CRT)  0% ventricular pacing (non CRT) | 17.02  (95%CI: 15.57-18.47) | 16.64  (95%CI: 15.19-18.09) | 0.350 | Y | N/A |
| Jamil AF | HFREF AF | CRT, PM, ICD | >95% BiV pacing (CRT)  0% ventricular pacing (non CRT) | 15.30  (95%CI: 13.75-16.86) | 14.22  (95%CI: 12.66-15.78) | 0.058 | Y | N/A |
| Kass GROUP 1 | HFpEF SR | Implantable cardiac device | AAIR/AAI | 12.47 (2.02)  (95%CI: 8.51-16.43)  (84%CI: 9.70-15.24) | 12.04 (1.74)  (95%CI: 8.63-15.45)  (84%CI: 9.66-14.42) | N/A | Y | Y |
| Kass GROUP 2 | HFpEF SR | Implantable cardiac device | AAIR/AAI | 13.09 (2.35)  (95%CI: 8.48-17.70)  (84%CI: 9.87-16.31) | 12.79 (2.14)  (95%CI: 8.60-16.98)  (84%CI: 9.86-15.72) | N/A | Y | Y |
| Passman | HFrEF SR | Dual chamber ICD | AAIR/VVI | 23.7 (6.1)  (95%CI: 11.74-35.66)  (84%CI: 15.34-32.06) | 23.8 (6.3)  (95%CI: 11.45-36.15)  (84%CI: 15.17-32.43) | 0.8 | Y | Y |
| Shanmugam | HF SR | CRT-PM/ICD | RAAVD/BiV | 16.1 (4.0)  (95%CI: 8.26-23.94)  (84%CI: 10.62-21.58) | 14.9 (3.7)  (95%CI: 7.65-22.15)  (84%CI: 9.83-19.97) | 0.024 | Y | Y |
| Sims | HFrEF SR | CRT | DDDR/DDD | 14.0 (3.2)  (95%CI: 7.73-20.28)  (84%CI: 9.62-18.38) | 13.9 (3.0)  (95%CI: 8.02-19.78)  (84%CI: 9.79-18.01) | 0.69 | Y | Y |
| Van Thielen | HFrEF SR | CRT | DDDR/DDD | 17.4 (0.7)  (95%CI: 16.03-18.77)  (84%CI: 16.44-18.36) | 17.8 (0.9)  (95%CI: 16.04-19.56)  (84%CI: 16.57-19.03) | 0.6 | Y | Y |
| **ET (secs)** | | | | | | | | |
| Jamil SR | HFrEF SR | CRT, PM, ICD | >95% BiV pacing (CRT)  0% ventricular pacing (non CRT) | 458.11  (95%CI: 390.33-525.89) | 465.10  (95%CI: 397.32-532.88) | 0.644 | Y | N/A |
| Jamil AF | HFREF AF | CRT, PM, ICD | >95% BiV pacing (CRT)  0% ventricular pacing (non CRT) | 417.23  (95%CI: 323.47-510.99) | 401.19  (95%CI: 307.43-494.95) | 0.395 | Y | N/A |
| Passman | HFrEF SR | Dual chamber ICD | AAIR/VVI | 946 (174)  (95%CI: 604.96-1287.04)  (84%CI: 707.62-1184.38) | 963 (168)  (95%CI: 633.72-1292.28)  (84%CI: 732.84-1193.16) | 0.38 | Y | Y |
| Shanmugam | HF SR | CRT-PM/ICD | RAAVD/BiV | 522 (192)  (95%CI: 145.68-898.32)  (84%CI: 258.96-785.04) | 474 (192)  (95%CI: 98.68-850.32)  (84%CI: 210.96-737.04) | 0.003 | Y | Y |
| **6MWT (m)** | | | | | | | | |
| Palmisano | HFrEF with drug refractory AF  AVJ Ablation and BiV pacing | CRT | VVIR/VVI | 267.2 (129.8)  (95%CI: 12.59-521.61)  (84%CI: 89.37-445.03) | 248.4 (127.9)  (95%CI: -2.28-499.08)  (84%CI: 73.18-423.63) | <0.001 | Y | Y |
| Pu | HFrEF SR | Three chamber CRT-PM/DF  Dual chamber PM | RAAVD LUV/BiV | 597 (85)  (95%CI: 430.4-763.6)  (84%CI: 480.55-713.45) | 563 (81)  (95%CI: 404.24-721.76)  (84%CI: 452.03-673.97) | 0.087 | Y | Y |
| Sims | HFrEF SR | CRT | DDDR/DDD | 376.8 (24.5)  (95%CI: 328.78-424.82)  (84%CI: 343.24-410.37) | 358.5 (40.7)  (95%CI: 278.73-438.27)  (84%CI: 302.74-414.26) | <0.05 | Y | Y |
| Zhao | HFrEF SR | Three-chamber PM  Dual chamber PM | RAAVD LUV/BiV | 502 (62)  (95%CI: 380.48-623.52)  (84%CI: 417.06-586.94) | 495 (63)  (95%CI: 371.52-618.48)  (84%CI: 408.69-581.31) | NS | Y | Y |

Atrial Fibrillation (AF); Biventricular (BiV); Cardiac Resynchronisation Therapy (CRT); Exercise Time (ET); Heart Failure (HF); Heart Failure preserved Ejection Fraction (HFpEF); Heart Failure reduced Ejection Fraction (HFrEF); Heart Rate (HR); Implantable Cardio-Defibrillator (ICD); Left Univentricular (LUV); Pace Maker (PM); Rate Adaptive Atrio-Ventricular Delay (RAAVD); Fixed Rate Pacing (RAPOFF); Standard Rate Adaptive Pacing (RAPON); Sinus Rhythm (SR); Oxygen Consumption/uptake (VO_2_); Six Minute Walk Test (6MWT)

Table S2 TLD RAPON vs RAP ON

| Study | Population & Rhythm | Device | Mode  (RAPON Tailored/ RAPON Standard) * | TLD RAPON  *Mean (SD)* | RAPON  *Mean (SD)* | P | 95% Overlap | 84% Overlap |
| --- | --- | --- | --- | --- | --- | --- | --- | --- |
| **Peak HR (bpm)** | | | | | | | | |
| Gierula | HFrEF  SR+AF | CRT | VVIR (AF)  DDDR (CRT)  AAIR (no CRT)  DDDR (no CRT + long AVD) | 98.6 (19.4)  (95%CI: 60.58-136.62)  (84%CI: 72.02-125.18) | 112.0 (20.3)  (95%CI: 72.21-151.79)  (84%CI: 84.19-139.81) | NS | Y | Y |
| Hsu | HFREF SR | CRT | CLS/DDDR | 110.7 (14.7)  (95%CI: 81.89-139.51)  (84%CI: 90.56-130.84) | 109.7 (14.1)  (95%CI: 82.06-137.34)  (84%CI: 90.38-129.02) | 0.67 | Y | Y |
| **Peak VO_2_ (ml/kg/min)** | | | | | | | | |
| Gierula | HFrEF  SR+AF | CRT | VVIR (AF)  DDDR (CRT)  AAIR (no CRT)  DDDR (no CRT + long AVD) | 16.72  (95%CI: 15.81-17.64) | 15.59  (95%CI: 14.47-16.43) | NS | Y | N/A |
| Hsu | HFREF SR | CRT | CLS/DDDR | 12.3 (4.9)  (95%CI: 2.70-21.90)  (84%CI: 5.59-19.01) | 12.9 (5.9)  (95%CI: 1.34-24.46)  (84%CI: 4.82-20.98) | 0.47 | Y | Y |
| Serova | HFpEF AF | Single chamber PM | VVIR/VVI | 14.3 (2.8)  (95%CI: 8.81-19.79)  (84%CI: 10.46-18.14) | 13.7 (3.2)  (95%CI: 7.43-19.97)  (84%CI: 9.31-18.09) | <0.001 | Y | Y |
| **ET (secs)** | | | | | | | | |
| Gierula | HFrEF  SR+AF | CRT | VVIR (AF)  DDDR (CRT)  AAIR (no CRT)  DDDR (no CRT + long AVD) | 483.15  (95%CI: 431.10-535.20) | 401.84  (95%CI: 363.62-458.07) | 0.044 | Y | N/A |
| Serova | HFpEF AF | Single chamber PM | VVIR/VVI | 567 (140)  (95%CI: 293.6-841.4)  (84%CI: 375.2-758.8) | 420 (175)  (95%CI: 77.0-763.0)  (84%CI: 180.25-659.75) | <0.0001 | Y | Y |
| **6MWT (m)** | | | | | | | | |
| Serova | HFpEF AF | Single chamber PM | VVIR/VVI | 423 (53)  (95%CI: 319.19-526.88)  (84%CI: 350.39-495.61) | 374 (97)  (95%CI: 183.88-564.12)  (84%CI: 241.11-506.89) | 0.003 | Y | Y |

Atrial Fibrillation (AF); Atrio-Ventricular Delay (AVD); Closed Loop Stimulation (CLS); Cardiac Resynchronisation Therapy (CRT); Exercise Time (ET); Heart Failure preserved Ejection Fraction (HFpEF); Heart Failure reduced Ejection Fraction (HFrEF); Heart Rate (HR); Pace Maker (PM); Standard Rate Adaptive Pacing (RAPON); Sinus Rhythm (SR); Tailored Rate Adaptive Pacing (TLD RAPON); Oxygen consumption/uptake (VO_2_); Six Minute Walk Test (6MWT)

Table S3. Risk of Bias in Randomised Control Trials

| **Study** | **Gierula** | **Pu** | **Serova** | **Zhao** |
| --- | --- | --- | --- | --- |
| **Domain 1:**  **Risk of bias arising from the randomisation process** | | | | |
| 1.1 Was the allocation sequence random? | Y | Y | Y | PY |
| 1.2 Was the allocation sequence concealed until participants were enrolled and assigned to interventions | Y | PY | Y | PY |
| 1.3 Did baseline differences between intervention groups suggest a problem with the randomisation process? | N | N | N | N |
| *Level of bias according to algorithm* | *Low Risk* | *Low Risk* | *Low Risk* | *Low Risk* |
| **Domain 2:**  **Risk of bias due to deviations from intended intervention** | | | | |
| ***For effect of assignment to intervention*** | | | | |
| 2.1 Were participants aware of their assigned intervention during the trial? | N | N | N | PN |
| 2.2 Were carers and people delivering the interventions aware of participants’ assigned intervention during the trial? | N | PN | PN | PN |
| 2.3 If Y/PY/NI to 2.1 or 2.2: Were there deviations from the intended intervention that arose because of the trial context? | N/A | N/A | N/A | N/A |
| 2.4 If Y/PY to 2.3: Were these deviations likely to have affected the outcome? | N/A | N/A | N/A | N/A |
| 2.5 If Y/PY/NI to 2.3: Were these deviations from intended intervention balanced between groups? | N/A | N/A | N/A | N/A |
| 2.6 Was an appropriate analysis used to estimate the effect of assignment to intervention? | Y | Y | Y | Y |
| 2.7 If N/PN/NI to 2.6: Was there potential for a substantial impact (on the result) of the failure to analyse participants in the group to which they were randomised? | N/A | N/A | N/A | N/A |
| *Level of bias according to algorithm* | *Low Risk* | *Low Risk* | *Low Risk* | *Low Risk* |
| ***For effect of adhering to intervention*** | | | | |
| 2.1 Were participants aware of their assigned intervention during the trial? | N | N | N | PN |
| 2.2 Were carers and people delivering the interventions aware of participants’ assigned intervention during the trial? | N | PN | PN | PN |
| 2.3 [if applicable] If Y/PY/NI to 2.1 or 2.2: Were important non-protocol-interventions balanced across intervention groups? | N/A | N/A | N/A | N/A |
| 2.4 [if applicable] Were there failures in implementing the intervention that could have affected the outcome? | N/A | N/A | N/A | N/A |
| 2.5 [if applicable] Was there non-adherence to the assigned intervention regimen that could have affected participant outcomes? | N/A | N/A | N/A | N/A |
| 2.6 If Y/PY/NI to 2.3, 2.4 or 2.5: Was an appropriate analysis used to estimate the effect of adhering to the intervention? | N/A | N/A | N/A | N/A |
| *Level of bias according to algorithm* | *Low Risk* | *Low Risk* | *Low Risk* | *Low Risk* |
| **Domain 3:**  **Risk of bias arising due to missing outcome data** | | | | |
| 3.1 Were data for this outcome available for all, or nearly all, participants randomised? | Y | Y | Y | Y |
| 3.2 If N/PN/NI to 3.1: Is there evidence that the result was not bias by missing outcome data? | N/A | N/A | N/A | N/A |
| 3.3 If N/PN to 3.2: Could missingness in the outcome depend on its true value? | N/A | N/A | N/A | N/A |
| 3.4 If Y/PY/NI to 3.3: Is it likely that missingness in the outcome depended on its true value? | N/A | N/A | N/A | N/A |
| *Level of bias according to algorithm* | *Low Risk* | *Low Risk* | *Low Risk* | *Low Risk* |
| **Domain 4:**  **Risk of bias in measurement of the outcome** | | | | |
| 4.1 Was the method of measuring the outcome inappropriate? | N | N | N | N |
| 4.2 Could measurement or ascertainment of the outcome have differed between intervention groups? | N | N | N | N |
| 4.3 If N/PN/NI to 4.1 or 4.2: Were outcome assessors aware of the intervention received by study participants? | N/A | N/A | N/A | N/A |
| 4.4 If Y/PY/NI to 4.3: Could assessment of the outcome have been influence by knowledge of intervention received? | N/A | N/A | N/A | N/A |
| 4.5 If Y/PY/NI to 4.4: Is it likely that assessment of the outcome was influence by knowledge of intervention received? | N/A | N/A | N/A | N/A |
| *Level of bias according to algorithm* | *Low Risk* | *Low Risk* | *Low Risk* | *Low Risk* |
| **Domain 5:**  **Risk of bias in selection of the reported result** | | | | |
| 5.1 Were the data that produced this result analysed in accordance with a pre-specified analysis plan that was finalised before unblinded outcome data were available for analysis? | Y | Y | Y | Y |
| 5.2 Is the numerical result being assessed likely to have been selected, on the basis of the results from multiple eligible outcome measurements (e.g. scales, definitions, time points) within the outcome domain? | N | N | N | N |
| 5.3 Is the numerical result being assessed likely to have been selected, on the basis of the results from multiple eligible analyses of the data? | N | N | N | N |
| *Level of bias according to algorithm* | *Low Risk* | *Low Risk* | *Low Risk* | *Low Risk* |

Yes (Y); probably yes (PY); not sure (NI); probably no (PN); no (N); Not Applicable (N/A)

Table S4. Risk of Bias for Crossover Trials

| **Study** | **Hsu** | **Jamil** | **Kass** | **Palmisano** | | | **Passman** | | **Shanmugam** | **Sims** | **Tse** | | **Van Thielen** |
| --- | --- | --- | --- | --- | --- | --- | --- | --- | --- | --- | --- | --- | --- |
| **Domain 1:**  **Risk of bias arising from the randomisation process in a crossover trial** | | | | | | | | | | | | | |
| 1.1 Was the allocation sequence random? | Y | Y | Y | Y | | | Y | | Y | Y | Y | | N |
| 1.2 Was the allocation sequence concealed until participants were enrolled and assigned to interventions | Y | Y | Y | Y | | | Y | | Y | Y | Y | | N/A |
| 1.3 Did baseline differences between intervention groups at the start of the first period suggest a problem with the randomisation process? | N | PN | PN | N | | | PN | | PN | PN | PN | | N/A |
| *Level of bias according to algorithm* | *Low Risk* | *Low Risk* | *Low Risk* | *Low Risk* | | | *Low Risk* | | *Low Risk* | *Low Risk* | *Low Risk* | | *High Risk* |
| **Domain S:**  **Risk of bias arising from period and carryover effects in a cross over trial** | | | | | | | | | | | | | |
| S.1 Was the number of participants allocated to each of the two sequences equal or early equal? | PY | PY | Y | Y | | | Y | | PY | PY | PY | NI | |
| S.2 If N/PN/NI to S.1: Were period effects accounted for in the analysis? | N/A | N/A | N/A | N/A | | | N/A | | N/A | N/A | N/A | NI | |
| S.3 Was there sufficient time for any carryover effects to have disappeared before outcome assessment in the second period? | PY | Y | Y | Y | | | Y | | Y | Y | Y | Y | |
| *Level of bias according to algorithm* | *Low Risk* | *Low Risk* | *Low Risk* | *Low Risk* | | | *Low Risk* | | *Low Risk* | *Low Risk* | *Low Risk* | *Moderate Risk* | |
| **Domain 2:**  **Risk of bias due to deviations from intended intervention in a crossover trial** | | | | | | | | | | | | | |
| ***For effect of assignment to intervention*** | | | | | | | | | | | | | |
| 2.1 Were participants aware of their assigned intervention during each period of the trial? | N | N | N | N | | | N | | N | N | N | PN | |
| 2.2 Were carers and people delivering the interventions aware of participants’ assigned intervention during each period of the trial? | N | N | N | N | | | N | | N | N | PN | PY | |
| 2.3 If Y/PY/NI to 2.1 or 2.2: Were there deviations from intended interventions that arose because of the trial context? | N/A | N/A | N/A | N/A | | | N/A | | N/A | N/A | N/A | N | |
| 2.4 If Y/PY to 2.3: Were these deviations likely to have affected the outcome? | N/A | N/A | N/A | N/A | | | N/A | | N/A | N/A | N/A | N/A | |
| 2.5 If Y/PY/NI to 2.3: Were there deviations from intended interventions balanced between interventions? | N/A | N/A | N/A | N/A | | | N/A | | N/A | N/A | N/A | N/A | |
| 2.6 Was an appropriate analysis used to estimate the effect of assignment to intervention? | Y | Y | Y | Y | | | Y | | Y | Y | Y | Y | |
| 2.7 If N/PN/NI to 2.6: Was there potential for a substantial impact (on the result) of the failure to analyse participants in the group to which they were randomised? | N/A | N/A | N/A | N/A | | | N/A | | N/A | N/A | N/A | N/A | |
| *Level of bias according to algorithm* | *Low Risk* | *Low Risk* | *Low Risk* | *Low Risk* | | | *Low Risk* | | *Low Risk* | *Low Risk* | *Low Risk* | *Low Risk* | |
| ***For effect of adhering to intervention*** | | | | | | | | | | | | | |
| 2.1 Were participants aware of their assigned intervention during each period of the trial? | N | N | N | N | | | N | | N | N | N | PN | |
| 2.2 Were carers and people delivering the interventions aware of participants’ assigned intervention during each period of the trial? | N | N | N | N | | | N | | N | N | PN | PY | |
| 2.3 [if applicable] If Y/PY/NI to 2.1 or 2.2: Were important non-protocol-interventions balanced between interventions? | N/A | N/A | N/A | N/A | | | N/A | | N/A | N/A | N/A | Y | |
| 2.4[if applicable] Were there failures in implementing the intervention that could have affected the outcome? | N/A | N/A | N/A | N/A | | | N/A | | N/A | N/A | N/A | N | |
| 2.5 [if applicable] Was there non-adherence to the assigned intervention regimen that could have affected participant outcomes? | N/A | N/A | N/A | N/A | | | N/A | | N/A | N/A | N/A | N | |
| 2.6 If Y/PY/NI to 2.3, 2.4 or 2.5: Was an appropriate analysis used to estimate the effect of adhering to the intervention? | N/A | N/A | N/A | N/A | | | N/A | | N/A | N/A | N/A | N/A | |
| *Level of bias according to algorithm* | *Low Risk* | *Low Risk* | *Low Risk* | *Low Risk* | | | *Low Risk* | | *Low Risk* | *Low Risk* | *Low Risk* | *Low Risk* | |
| **Domain 3:**  **Risk of bias arising due to missing outcome data in a crossover trial** | | | | | | | | | | | | | |
| 3.1 Were data for this outcome available for all, or nearly all, participants randomised? | Y | Y | Y | Y | | Y | | Y | | Y | Y | Y | |
| 3.2 If N/PN/NI to 3.1: Is there evidence that the results was not bias by missing outcome data? | N/A | N/A | N/A | N/A | | N/A | | N/A | | N/A | N/A | N/A | |
| 3.3 If N/PN to 3.2: Could missingness in the outcome depend on its true value? | N/A | N/A | N/A | N/A | | N/A | | N/A | | N/A | N/A | N/A | |
| 3.4 If Y/PY/NI to 3.3: Is it likely that missingness in the outcome depended on its true value? | N/A | N/A | N/A | N/A | | N/A | | N/A | | N/A | N/A | N/A | |
| *Level of bias according to algorithm* | *Low Risk* | *Low Risk* | *Low Risk* | *Low Risk* | | *Low Risk* | | *Low Risk* | | *Low Risk* | *Low Risk* | *Low Risk* | |
| **Domain 4:**  **Risk of bias in measurement of the outcome in a crossover trial** | | | | | | | | | | | | | |
| 4.1 Was the method of measuring the outcome inappropriate? | N | N | N | N | N | | | N | | N | N | N | |
| 4.2 Could measurement or ascertainment of the outcome have differed between interventions within each sequence? | N | N | N | N | N | | | N | | N | N | N | |
| 4.3 If N/PN/NI to 4.1 or 4.2: Were outcome assessors aware of the intervention received by study participants? | N/A | N/A | N/A | N/A | N/A | | | N/A | | N/A | N/A | N/A | |
| 4.4 If Y/PY/NI to 4.3: Could assessment of the outcome have been influence by knowledge of intervention received? | N/A | N/A | N/A | N/A | N/A | | | N/A | | N/A | N/A | N/A | |
| 4.5 If Y/PY/NI to 4.4: Is it likely that assessment of the outcome was influence by knowledge of intervention received? | N/A | N/A | N/A | N/A | N/A | | | N/A | | N/A | N/A | N/A | |
| *Level of bias according to algorithm* | *Low Risk* | *Low Risk* | *Low Risk* | *Low Risk* | *Low Risk* | | | *Low Risk* | | *Low Risk* | *Low Risk* | *Low Risk* | |
| **Domain 5:**  **Risk of bias in selection of the reported result in a crossover trial** | | | | | | | | | | | | | |
| 5.1 Were the data that produced this result analysed in accordance with a pre-specified analysis plan that was finalised before unblinded outcome data were available for analysis? | Y | Y | Y | Y | Y | | | Y | | Y | Y | Y | |
| 5.2 Is the numerical result being assessed likely to have been selected, on the basis of the results from multiple eligible outcome measurements (e.g. scales, definitions, time points) within the outcome domain? | N | N | N | N | N | | | N | | N | N | N | |
| 5.3 Is the numerical result being assessed likely to have been selected, on the basis of the results from multiple eligible analyses of the data? | N | N | N | N | N | | | N | | N | N | N | |
| 5.4 Is the result based on data from both periods sought? But unavailable on the basis of carryover having been identified? | N | N | N | N | N | | | N | | N | N | N | |
| *Level of bias according to algorithm* | *Low Risk* | *Low Risk* | *Low Risk* | *Low Risk* | *Low Risk* | | | *Low Risk* | | *Low Risk* | *Low Risk* | *Low Risk* | |

Yes (Y); probably yes (PY); not sure (NI); probably no (PN); no (N); Not Applicable (N/A)

Tabel S5 MeSH Terms

|  | **Search Term** | **MeSH Terms** |
| --- | --- | --- |
| **1** | **(Rate Adaptive Pacing)** | **Rate.** "J Rehabil Assist Technol Eng"[Journal:__jid101671667] OR "rate"[All Fields]  **Adaptive:** "acclimatization"[MeSH Terms] OR "acclimatization"[All Fields] OR "adaptation"[All Fields] OR "adaptations"[All Fields] OR "adapt"[All Fields] OR "adaptabilities"[All Fields] OR "adaptability"[All Fields] OR "adaptable"[All Fields] OR "adaptational"[All Fields] OR "adaptative"[All Fields] OR "adapte"[All Fields] OR "adapted"[All Fields] OR "adapting"[All Fields] OR "adaption"[All Fields] OR "adaptions"[All Fields] OR "adaptive"[All Fields] OR "adaptively"[All Fields] OR "adaptiveness"[All Fields] OR "adaptivity"[All Fields] OR "adapts"[All Fields]  **Pacing:** "paced"[All Fields] OR "paces"[All Fields] OR "pacing"[All Fields] OR "pacings"[All Fields] |
| **2** | **Exercise Testing** | "exercise test"[MeSH Terms] OR ("exercise"[All Fields] AND "test"[All Fields]) OR "exercise test"[All Fields] OR ("exercise"[All Fields] AND "testing"[All Fields]) OR "exercise testing"[All Fields] |
| **3** | **Heart Failure** | ("heart failure"[MeSH Terms] OR ("heart"[All Fields] AND "failure"[All Fields]) OR "heart failure"[All Fields]) |
| **4** | **Heart Rate** | "heart rate"[MeSH Terms] OR ("heart"[All Fields] AND "rate"[All Fields]) OR "heart rate"[All Fields] |
| **5** | **(Chronotropic Incompetence)** | **Chronotropic:** "chronotropic"[All Fields] OR "chronotropically"[All Fields] OR "chronotropism"[All Fields]  **Incompetence:** "incompetence"[All Fields] OR "incompetences"[All Fields] OR "incompetency"[All Fields] OR "incompetent"[All Fields] OR "incompetents"[All Fields] |
| **6** | **(Exercise Capacity)** | **Exercise:** "exercise"[MeSH Terms] OR "exercise"[All Fields] OR "exercises"[All Fields] OR "exercise therapy"[MeSH Terms] OR ("exercise"[All Fields] AND "therapy"[All Fields]) OR "exercise therapy"[All Fields] OR "exercise's"[All Fields] OR "exercised"[All Fields] OR "exerciser"[All Fields] OR "exercisers"[All Fields] OR "exercising"[All Fields]  **Capacity:** "capacities"[All Fields] OR "capacity"[All Fields] |
| **7** | **(Exercise Intolerance)** | **Exercise:** "exercise"[MeSH Terms] OR "exercise"[All Fields] OR "exercises"[All Fields] OR "exercise therapy"[MeSH Terms] OR ("exercise"[All Fields] AND "therapy"[All Fields]) OR "exercise therapy"[All Fields] OR "exercise's"[All Fields] OR "exercised"[All Fields] OR "exerciser"[All Fields] OR "exercisers"[All Fields] OR "exercising"[All Fields]  **Intolerance:** "intolerabilities"[All Fields] OR "intolerability"[All Fields] OR "intolerable"[All Fields] OR "intolerably"[All Fields] OR "intolerance"[All Fields] OR "intolerances"[All Fields] OR "intolerant"[All Fields] OR "intolerants"[All Fields] |
| **8** | **Cardiac Resynchronisation Therapy** | "cardiac resynchronization therapy"[MeSH Terms] OR ("cardiac"[All Fields] AND "resynchronization"[All Fields] AND "therapy"[All Fields]) OR "cardiac resynchronization therapy"[All Fields] OR ("cardiac"[All Fields] AND "resynchronisation"[All Fields] AND "therapy"[All Fields]) OR "cardiac resynchronisation therapy"[All Fields] |
| **9** | **(Cardio-Defibrillator)** | **Cardio:** "heart"[MeSH Terms] OR "heart"[All Fields] OR "cardio"[All Fields]  **Defibrillator:** "defibrilator"[All Fields] OR "defibrillate"[All Fields] OR "defibrillated"[All Fields] OR "defibrillates"[All Fields] OR "defibrillating"[All Fields] OR "defibrillations"[All Fields] OR "defibrillator's"[All Fields] OR "defibrillators"[MeSH Terms] OR "defibrillators"[All Fields] OR "defibrillator"[All Fields] OR "electric countershock"[MeSH Terms] OR ("electric"[All Fields] AND "countershock"[All Fields]) OR "electric countershock"[All Fields] OR "defibrillation"[All Fields] |
| **10** | **Pacemaker:** | "pacemaker's"[All Fields] OR "pacemaker, artificial"[MeSH Terms] OR ("pacemaker"[All Fields] AND "artificial"[All Fields]) OR "artificial pacemaker"[All Fields] OR "pacemaker"[All Fields] OR "pacemakers"[All Fields] OR "pacemaking"[All Fields] |
| **11** | **Full Text; Humans; English** | fft[Filter]: loattrfull text[subset]  humans[Filter]: humans[MH]  english[Filter]: english [LA] |

Table S6 Search Strategy

|  | **Search strategy** |
| --- | --- |
| **1** | 1 AND 11 |
| **2** | 1 AND 2 AND 11 |
| **3** | 1 AND 2 AND 3 AND 11 |
| **4** | 1 AND 3 AND 11 |
| **5** | 1 AND 3 AND 4 AND 11 |
| **6** | 1 AND 3 AND 4 AND 5 AND 11 |
| **7** | 1 AND 3 AND 4 AND 5 AND 6 OR 7 AND 11 |
| **8** | 1 AND 2 AND 3 AND 8 OR 9 OR 10 AND 11 |
